# Improvement of Error Correction in Nonequilibrium Information Dynamics

**DOI:** 10.3390/e25060881

**Published:** 2023-05-31

**Authors:** Qian Zeng, Ran Li, Jin Wang

**Affiliations:** 1 State Key Laboratory of Electroanalytical Chemistry, Changchun Institute of Applied Chemistry, Changchun 130022, China; 2 Center for Theoretical Interdisciplinary Sciences, Wenzhou Institute, University of Chinese Academy of Sciences, Wenzhou 325001, China; 3 Department of Chemistry and Physics, State University of New York, Stony Brook, NY 11794, USA

**Keywords:** nonequilibrium information dynamics, error correction, entropy production rate

## Abstract

Errors are inevitable in information processing and transfer. While error correction is widely studied in engineering, the underlying physics is not fully understood. Due to the complexity and energy exchange involved, information transmission should be considered as a nonequilibrium process. In this study, we investigate the effects of nonequilibrium dynamics on error correction using a memoryless channel model. Our findings suggest that error correction improves as nonequilibrium increases, and the thermodynamic cost can be utilized to improve the correction quality. Our results inspire new approaches to error correction that incorporate nonequilibrium dynamics and thermodynamics, and highlight the importance of the nonequilibrium effects in error correction design, particularly in biological systems.

## 1. Introduction

In information processing and transfer, the transmission of messages through communication channels is often impaired by unwanted noise. As a result, it is inevitable that the useful information carried by these messages will experience some loss during transmission. The channel coding principle, developed by R. W. Hamming [1], C. E. Shannon [2], R. G. Gallager [3], and A. J. Viterbi [4] states that no matter how we encode the messages, it is impossible to transmit them at a rate above the information capacity with zero error probability through noisy channels. Therefore, to reduce errors, we must either decrease the transmission rate or improve the quality of the channels. Error correction coding methods have been developed based on these theories to minimize the loss of useful information at the information receiver end.

In biological systems, error correction is a crucial process that plays an essential role in maintaining the fidelity and accuracy of biological information transfer [5,6,7]. For example, in DNA replication, error correction mechanisms help to identify and correct errors that occur during the copying of DNA molecules, while in protein synthesis, error correction mechanisms ensure that the correct amino acids are incorporated into the growing polypeptide chain, minimizing the occurrence of misfolded or non-functional proteins. Therefore, investigating error correction from the perspective of statistical physics and biophysics is important in understanding the underlying principles of these processes.

In information theory, error correction methodologies are widely used to ensure the quality of information transmission in noisy communication channels [8,9,10,11,12,13,14,15]. While these techniques are often studied in an engineering context [16,17,18,19,20,21], the underlying physics is not always clear. Information processing and communication are not isolated events but should be considered as open systems that exchange energy and information with their environments, which can have a significant impact. The complexity and stochasticity of these processes, along with environmental influences, make information transmission a nonequilibrium process. In this context, different symbols in messages can be distorted by channels with varying error probabilities during transmission, resulting in nonequilibrium behavior of the received messages in channel models. Understanding the relationship between the error correction in decoding and the nonequilibrium in transmission is crucial, but there is still a lack of studies exploring this connection.

The memoryless channel model is a critical component in the fields of information and communication theories [22,23]. This study focuses on investigating the impact of nonequilibrium dynamics and thermodynamics on error corrections in the memoryless channel model. We establish the Markovian information dynamics [24,25,26] of this channel with block inputs and outputs and quantify the nonequilibrium of the memoryless channel models by calculating the difference between their transmission probabilities. This nonequilibrium measures the strength of the information flux or nonequilibrium information driving force in information transmission. We prove that the performance of error correction at the decoder can be analytically expressed as a function of the nonequilibrium of these models. Our analysis reveals that error correction performance, characterized by the upper bound of the error probability, is a convex function of the nonequilibrium in information transmission, with the unique maximum error probability being 1 at the equilibrium point of the information dynamics. Additionally, the upper bound monotonically decreases as the nonequilibrium increases. Moreover, we found that the dissipation cost in information transmission, characterized by the entropy production rate (EPR) [27,28,29,30,31,32,33,34,35,36,37,38,39,40], increases as the nonequilibrium increases. We discovered that the EPR can be used to measure the reliability of the information transmission, indicating that the thermodynamic cost can be utilized to enhance the quality of information transmission and reduce the decoding error probability. Our findings inspire us to develop novel approaches for error correction based on nonequilibrium dynamics and thermodynamics. We tested our conclusions using a simple binary memoryless channel model, and the numerical results supported our findings. Finally, we discuss the potential applications of our conclusions in biological systems.

## 2. Memoryless Channel Model and Error Correction

### 2.1. Memoryless Channel Model and Block Encoding

We start with the memoryless channel model to explore error correction. The basic idea behind any error correction strategy is to encode information redundantly within a larger system in order to mitigate the impact of errors and prevent information loss.

An information sender transmits random symbols *s* which form a set S. To reduce the error during the transmission in the noisy channel, each symbol *s* can be encoded by an encoder at the sender end into a block code of length *N*, $X_{s}=\left\{x_{1},x_{2},\cdots ,x_{N}\right\}$ consisting of the letters x=0,1,..,n−1 (*n* is the total number of *x*). This is known as the fixed block encoding strategy [2]. The set of all the input block codes $X_{s}$ is denoted by X. However, due to the noisy channels, the receiver may receive a noisy output block $Y=\{y_{1},y_{2},\cdots ,y_{N}\}$ from the channel with output letters y=0,1,..,n−1. The set of all the output block codes *Y* is denoted by Y. In this process, the rate of transmission is then determined by the length of each block code, i.e., R=1/N.

For general memoryless channels, we assume that the channel corrupts the symbols *s* independently according to different noise distributions, respectively. Determined by the noises, the random mapping between the input letters *x* in the input block and the output letters *y* in the output block can be described by the transmission probabilities, q(y|x), which quantify the conditional probabilities that one receives the letter *y* when the letter *x* is sent. Since the channel is memoryless, the total transmission probability of the output block *Y* when the input block $X_{s}$ (s∈S) with length of *N* is sent can be written as

(1) $$\begin{array}{c} q_{N}\left(Y|X_{s}\right)=\prod_{i=1}^{N}q\left(y_{i}|x_{i}\right). \end{array}$$

In fact, the input and output letters in most general channels are continuously valued. However, the essence of the information transfer problem is that it is essentially discrete. Therefore, we will concentrate on discrete memoryless channels in this work, without the loss of generality.

### 2.2. Error Correction Decoding

In general, if we let the length of the block codes $X_{s}$ be some number *N*, then there exist $n^{N}$ possible block codes (*n* is the total number of the letters *x*), which can be assigned to the symbols *s* at the encoder. To decode the received noisy blocks correctly, the assignment of $X_{s}$ to each *s* should be consistent with the a priori knowledge of the noise or transmission probability distributions q(y|x). Then, we have to set up a strategy, the so-called “error correction”, to map the noisy output blocks *Y* back into the space of symbols of the information source S.

A simple decoding rule is to choose the decoding result *s* for which

(2) $$\begin{array}{c} q_{N}\left(Y|X_{s}\right)\geq q_{N}\left(Y|X_{s^{\prime }}\right),\;\mathrm{for}\;\mathrm{all}\;s\neq s^{\prime }. \end{array}$$

This means to choose a symbol *s* from the set S that maximizes the transmission probability or likelihood $q_{N}\left(Y|X_{s}\right)$ (given in Equation (1)) of the received block corresponding to the original sent block rather than other ones $s^{\prime }\neq s$.

According to the decoding rule given in Equation (2), the block codes $X_{s}$ should be carefully chosen such that the space of the output blocks Y can be divided into *M* (*M* is the total number of the symbols) mutually disjointing decoding subsets $Y_{s}$ corresponding to each transmitted symbol *s*, i.e.,

(3) $$\begin{array}{c} Y_{s}⋂Y_{s^{\prime }}=Ø\;\mathrm{for}\;s^{\prime }\neq s,\;\mathrm{and}\;\overset{M}{\underset{i=1}{⋃}}Y_{s_{i}}\subseteq Y. \end{array}$$

Due to the decoding rule in Equation (2), the transmission probability of each $Y\in Y_{s}$ corresponding to $X_{s}$, given by Equation (1), should be greater than that of *Y* conditioning on another input block $X_{s^{\prime }}$ for $s^{\prime }\neq s$. The reason behind this approach is straightforward: by selecting each input block code $X_{s}$ in a manner that is consistent with the a priori knowledge of the transmission probabilities, we can decode any output block *Y* easily. Specifically, decoding *Y* as belonging to $Y_{s}$ yields symbol *s*, while decoding *Y* as belonging to $Y_{s^{\prime }}$ yields symbol $s^{\prime }$. Any other decoding scheme, such as decoding $Y\in Y_{s^{\prime }}$ as *s*, is more prone to errors.

As an example, we consider the binary memoryless channel. The transmitted symbols are given as s=a,b and the encoding letters given as x=0,1. Assume that the transmission probabilities satisfy the following conditions

$$\begin{array}{c} q(y=0|x=0)>1/2\;\;\;,q(y=1|x=0)=1-q(y=0|x=0)<1/2\\ q(y=0|x=1)<1/2\;\;\;,q(y=1|x=1)=1-q(y=0|x=1)>1/2 \end{array}$$

By using the block codes of length 3, we can encode the symbol *a* into the block $X_{a}=000$ and *b* into the block $X_{b}=111$. We then have the set of the input block codes X={000,111} (N=3) which can be sent into the channel. This encoding method guarantees that the output blocks is separated into two disjoint subsets.

Distorted by the noisy channels, there are $2^{3}=8$ possible output blocks *Y* of length 3 in total, and these blocks form an output set Y={000,001,010,011,100,101,110,111}. Because the transmission probabilities q(y=0|x=1)<1/2 and q(y=0|x=0)>1/2, there are usually less than half of the letters x=0 distorted by the noises when the block “000” corresponding to *a* is transmitted. In addition, since q(y=1|x=1)>1/2 and q(y=1|x=0)<1/2, there are usually less than half of the letters x=1 distorted by the noises when “111” corresponding to *b* is transmitted. In this way, the output set Y is separated into two subsets, $Y_{a}=\left\{000,001,010,100\right\}$ and $Y_{b}=\left\{011,101,110,111\right\}$, that satisfy the condition given by Equation (3).

Thus, according to the decoding rule of maximizing the transmission probability, we simply decode the output blocks within the decoding set $Y_{a}=\left\{000,001,010,100\right\}$ into *a* and decode the blocks in the decoding set $Y_{b}=\left\{011,101,110,111\right\}$ into *b*. If we make the block length *N* larger (the rate of transmission R=1/N then becomes smaller), then the law of large number works and we can make the transmission more accurately with less error probability [22].

### 2.3. Random Encoding and Decoding Strategy

Although the fixed block encoding strategies can be effective in numerous scenarios, it is important to acknowledge that designing the fixed block codes can be challenging. Alternatively, the random encoding strategies may be more convenient in many cases, and they can achieve similar performance compared to the fixed block encoding [1].

For the random encoding strategies, we can encode the original symbols *s* with the random input blocks $X_{s}$ according to an input distribution $Q_{N}\left(X\right)$, and still decode the output block *Y* according to Equations (2) and (3). Here, the random choice of $X_{s}$ can be implemented by randomly choosing the letters *x* in a block independently according to an identical input distribution Q(x) such that the probability of a block $X_{s}=\left\{x_{1},x_{2},\cdots ,x_{N}\right\}$ can be given by

(4) $$\begin{array}{c} Q_{N}\left(X_{s}\right)=\prod_{i}^{N}Q\left(x_{i}\right). \end{array}$$

We now explain how to encode and decode the information by using the random encoding strategy for the binary memoryless channel in the last subsection. If we use the random encoding method instead in this example with the same settings in the above, and if the input letters x=0,1 are chosen randomly with equal probability, i.e., Q(x=0)=Q(x=1)=1/2, then all the possible input blocks can be chosen from the input set X={000,001,010,011,100,101,110,111} with the same probability ${\left(1/2\right)}^{3}=1/8$. A possible assignment of the blocks for each symbols can be $X_{a}=000$ and $X_{b}=111$, with the same output decoding sets shown in the above. Alternatively, another possible assignment can be $X_{a}=011$ and $X_{b}=001$. Consequentially, the output decoding set for the symbol *a* turns out to be $Y_{a}=\left\{010,011,110,111\right\}$; and the decoding set for the symbol *b* becomes $Y_{b}=\left\{000,001,100,101\right\}$.

### 2.4. Performance of Error Correction

Although we can reduce data transmission errors by elaborating the encoder or increasing the block length *N*, there is still a risk of decoding errors, if the input block $X_{s}$ is corrupted and transformed into an output block *Y* that does not belong to the intended subset $Y_{s}$, but instead belongs to another subset $Y_{s^{\prime }}$ ($s^{\prime }\neq s$). In this case, the block *Y*, which was originally associated with symbol *s*, may be mistakenly decoded into $s^{\prime }$. Therefore, it is necessary to estimate the error probabilities of decoding in order to quantify the performance of error correction.

In general, the error probability is a non-trivial function that requires sophisticated methods to calculate accurately. However, it may be more practical to estimate a simple upper bound of the error probability, which is often sufficiently close to the true value. Reducing this upper bound on the error probability is likely to improve error correction performance.

By randomly selecting code blocks $X_{s}$ of length *N* in accordance with the input distribution outlined in Equation (4) and applying the decoding rules specified in Equations (2) and (3), we can determine the upper bound of the average error probability associated with decoding any transmitted symbol *s* as a symbol $s^{\prime }$ that differs from *s*. An upper bound is given by [19]

(5) $$\begin{array}{ccc} P_{e,s} & \leq & {\left(M-1\right)}^{\rho -1}U\left(Q,q_{N},\rho \right),\;\mathrm{for}\;\rho \in \left[1,2\right], \end{array}$$

with

(6) $$\begin{array}{ccc} U(Q,q_{N},\rho ) & = & \sum_{Y}{\left[\sum_{X}Q_{N}\left(X\right){\left[q_{N}\left(Y|X\right)\right]}^{\frac{1}{\rho }}\right]}^{\rho },\;\mathrm{for}\;\mathrm{general}\;\mathrm{channels}\\ & = & {\left\{\sum_{y}{\left[\sum_{x}Q\left(x\right){\left[q\left(y|x\right)\right]}^{\frac{1}{\rho }}\right]}^{\rho }\right\}}^{N},\;\mathrm{for}\;\mathrm{memoryless}\;\mathrm{channels} \end{array}$$

where M≥2 is the total number of the possible transmitted symbols *s*; $Q_{N}\left(X\right)=\prod_{i}Q\left(x_{i}\right)$ is the arbitrary input distribution of all the possible input block codes *X* as described in Equation (4); $q_{N}\left(y|x\right)$ is the transmission probabilities of the channel with respect to the block codes.

If the channel is memoryless, then $q_{N}\left(y|x\right)$ can be reduced into the products of the transmission probabilities with respect to the letters in a block as shown in Equation (1). This gives rise to the second equality in Equation (6) for the memoryless channels on average. Here, “average” means to estimate the error probability over the ensemble of the block codes *X* independently of any concrete assignment of the blocks for the symbols.

When the parameter ρ is chosen carefully, the bound in Equation (5) can be surprisingly close to the true value. The importance of this inequality is that it can be used as an estimate of the highest error probability or the worst performance of error correction under a given condition. A smaller upper bound always means better performance.

It can be seen that the upper bound in Equation (5) depends on both the input distribution Q(x) and the transmission probabilities $q_{N}\left(Y|X_{s}\right)$ or q(y|x) of the channel. In practice, this observation provides two alternative methods to reduce the upper bound of error probability and to improve the performance of error correction. Note that q(y|x) is the characterization of the memoryless channel and it mainly depends on the environments of the channel. For the information channels which are too large to control, such as the classical communication systems, the related studies mainly focus on the optimization of the input distribution Q(x) to reduce the upper bound of the averaged error probabilities for a better performance of the error correction. However, for the small systems where the channels are small enough to control, the input distributions may almost totally depend on the systems themselves, such as the DNA in a cell and the neural network in the brain. It is then possible to optimize the transmission probabilities q(y|x) through the control on the channel via either genetic and epigenetic modulations for better information transmission, or, say, for improving the reliability of the error correction in the systems.

### 2.5. Concavity of Upper Bound of Error Correction

For the purpose of the transmission probability or channel control, we will prove in the following that the worst performance of the error correction characterized by the upper bound in Equation (5) is a concave function of the transmission probability distribution $q_{N}\left(Y|X\right)$, given a fixed block length *N*, input distribution $Q_{N}\left(X\right)$, and parameter ρ. This finding underscores the worst-case performance of error correction, highlighting the need to optimize the transmission probabilities to enhance error correction.

We first show that all the possible transmission probability distributions $q_{N}$ form a convex set $Q_{N}=\left\{q_{N}:q_{N}\left(Y|X\right)\geq 0,\sum_{Y}q_{N}\left(Y|X\right)=1\right\}$. Here, the constraints on $Q_{N}$ are consistent with the nonnegativity and normalization of the conditional probability distribution. To see the convexity of $Q_{N}$, we choose arbitrarily two transmission probability distributions $q_{N}^{\left(1\right)},q_{N}^{\left(2\right)}\in Q_{N}$. Consider the convex combination at each output *Y* in the following form

$$\begin{array}{c} q_{N}^{\left(\lambda \right)}\left(Y|X\right)=\lambda q_{N}^{\left(1\right)}\left(Y|X\right)+\left(1-\lambda \right)q_{N}^{\left(2\right)}\left(Y|X\right)\;,\;\;\;\lambda \in \left[0,1\right]\;. \end{array}$$

One can easily check that both the constraints, nonnegativity and normalization, are satisfied by $q_{N}^{\left(\lambda \right)}$ for all *Y* at each *X* because $q_{N}^{\left(\lambda \right)}\left(Y|X\right)\geq 0$ and $\sum_{Y}q_{N}^{\left(\lambda \right)}\left(Y|X\right)=\lambda \sum_{Y}q_{N}^{\left(1\right)}\left(Y|X\right)+\left(1-\lambda \right)\sum_{Y}q_{N}^{\left(2\right)}\left(Y|X\right)=1$. This indicates that $q_{N}^{\left(\lambda \right)}$ is also a transmission probability distribution, which is contained in $Q_{N}$. Then, the convexity of $Q_{N}$ can be verified directly by using the definition of the convex set.

Then, we introduce the function

$$\begin{array}{c} ∥q_{\epsilon }{∥}_{Y}={\left[\sum_{X}{\left[q_{\epsilon }\left(Y|X\right)\right]}^{\frac{1}{\rho }}\right]}^{\rho } \end{array}$$

which can be recognized as the $\frac{1}{\rho }$ norm of the function $q_{\epsilon }$ at each *Y* (the norm sums up the *X* in $q_{\epsilon }$, and ϵ means ‘error’). One can show that the function *U* in the upper bound in Equation (5) can be rewritten as

$$\begin{array}{c} U=\sum_{Y}{∥q_{\epsilon }∥}_{Y} \end{array}$$

where $q_{\epsilon }\left(Y|X\right)={\left[Q_{N}\left(X\right)\right]}^{\rho }q_{N}\left(Y|X\right)$. It should be noted that, with fixed *N*, $Q_{N}\left(X\right)$, and ρ, the Minkowski inequality of the norms for ρ∈[1,2] guarantees the concavity of the function *U* on the convex set $Q_{N}$. By using the definition of the concave functions [41], one can show

(7) $$\begin{array}{ccc} \lambda U\left(q_{N}^{\left(1\right)}\right)+\left(1-\lambda \right)U\left(q_{N}^{\left(2\right)}\right) & = & \lambda \sum_{Y}∥q_{\epsilon }^{\left(1\right)}{∥}_{Y}+\left(1-\lambda \right)\sum_{Y}{∥q_{\epsilon }^{\left(2\right)}∥}_{Y}\\ & \leq & \sum_{Y}{∥\lambda q_{\epsilon }^{\left(1\right)}+\left(1-\lambda \right)q_{\epsilon }^{\left(2\right)}∥}_{Y}\\ & = & \sum_{Y}{\left[\sum_{X}Q_{N}\left(X\right){\left[\lambda q^{\left(1\right)}\left(Y|X\right)+\left(1-\lambda \right)q^{\left(2\right)}\left(Y|X\right)\right]}^{\frac{1}{\rho }}\right]}^{\rho }\\ & = & U(q_{N}^{\left(\lambda \right)}), \end{array}$$

where the Minkowski inequality of the norms for ρ∈[1,2] is used in the second line. Since the prefactor ${\left(M-1\right)}^{\rho -1}$ in Equation (5) is a positive constant (if ρ is fixed), then the upper bound of the error probability is always a concave function of the transmission probability distribution $q_{N}$. Note that in the present work we use the conventional rules that the concave function is the concave down function with a negative second derivative and the convex function is the concave up function with a positive second derivative.

Furthermore, the upper bound achieves the unique maximum ${\left(M-1\right)}^{\rho -1}$ when $q_{N}\left(Y|X_{s^{\prime }}\right)=q_{N}\left(Y|X_{s}\right)$ for all $s^{\prime }\neq s$. Consequentially, we have that $P_{N}\left(Y\right)=q_{N}\left(Y|X\right)$ for all *s*. This result has an intuitive explanation: the output *Y* is totally independent of the input $X_{s}$ and hence is independent of the transmitted symbol *s*, so that arbitrary *s* cannot be distinguished from another $s^{\prime }$ at the decoder. This leads to the worst performance of the error correction where the error occurs almost with probability 1 no matter which input distribution Q(x) is chosen.

For the memoryless channels, the upper bound of the error probability can be given by the second equality in Equation (6), $U=u^{N}$, where $u=\sum_{y}{\left[\sum_{x}Q\left(x\right){\left[q\left(y|x\right)\right]}^{\frac{1}{\rho }}\right]}^{\rho }$ can be regarded as the function *U* for the blocks of length N=1, and q(y|x) is the transmission probability of the channel for the letters in the blocks. Thus, *u* is concave on the set of q(y|x), denoted by Q, and Q is a convex set, according to the proof in Equation (7). Then, the worst performance of a memoryless channel can happen with error probability 1 when $q\left(y|x\right)=q\left(y|x^{\prime }\right)$ for all $x^{\prime }\neq x$.

In summary, we have demonstrated the concavity of the upper bound of error correction. Note that the environments of the channel are inherently complex. The nonequilibrium that arises from this complexity can influence both the information transmission and the error correction significantly. With the perspective of the nonequilibrium information dynamics and thermodynamics, we will investigate the relationship between the performance of the error correction and the channel-induced nonequilibrium in the following sections. Specifically, we will show that the worst performance of the error correction, where the error probability is almost 1 and occurs in the equilibrium state of the memoryless channel, where the output is independent of the input completely.

## 3. Nonequilibrium Information Dynamics

### 3.1. Information Dynamics of Memoryless Channel

To introduce a dynamical description to the memoryless channel model, it is reasonable to assume that the channel needs time to deal with the codes, i.e., the sender transfers the blocks $X_{t-1}$ at time t−1 and the receiver gets the outputs $Y_{t}$ at time *t*, where the processing time is assumed to be unit. On the other hand, if the random encoding method is employed, the correspondence between the block code *X* and the transmitted symbol *s* is not necessarily one to one. To simplify the discussion without losing the generality, we will not delve into a detailed discussion on assignments of the block codes for the transmitted symbols. Instead, we assume that the input blocks *X* are independently generated according to an identical input distribution $Q_{N}\left(X\right)$, where *N* is the length of the block codes. In addition, each letters *x* within an input block can be independently chosen based on an identical input distribution Q(x). The relationship between $Q_{N}\left(X\right)$ and Q(x) is provided in Equation (4).

In the context of information dynamics, we are interested in understanding how the input and output of the channel evolve in time together. We use the notation $Z_{t}=\left(X_{t},Y_{t}\right)$, which is the composition of the input block $X_{t}$ and output block $Y_{t}$, to represent the information state of the channel model. Due to the dynamical description in the above, the input $X_{t}$, generated following the distribution $Q_{N}\left(X_{t}\right)$, is independent of both $X_{t-1}$ and $Y_{t}$, and the output $Y_{t}$ merely depends on the previous input $X_{t-1}$ according to the transmission probability $q_{N}\left(Y_{t}|X_{t-1}\right)$. In addition, both $X_{t}$ and $Y_{t}$ are independent of $Y_{t-1}$. Therefore, the correlation between the information state $Z_{t-1}=\left(X_{t-1},Y_{t-1}\right)$ and the successive state $Z_{t}=\left(X_{t},Y_{t}\right)$ in time follows a Markovian connection. The transition probability from $Z_{t-1}$ to $Z_{t}$ can be given by

(8) $$\begin{array}{c} P\left(Z_{t}|Z_{t-1}\right)=Q_{N}\left(X_{t}\right)q_{N}\left(Y_{t}|X_{t-1}\right). \end{array}$$

With this transition probability, the evolution of the distribution $P(Z_{t})$ follows the Markovian information dynamics as:

(9) $$\begin{array}{c} P\left(Z_{t}\right)=\sum_{Z_{t-1}}P\left(Z_{t}|Z_{t-1}\right)P\left(Z_{t-1}\right). \end{array}$$

It is recognized that $P\left(Z_{t\to \infty }\right)=P\left(Z\right)=Q_{N}\left(X\right)P_{N}\left(Y\right)$ with $P_{N}\left(Y\right)=\sum_{X}Q_{N}\left(X\right)q_{N}\left(Y|X\right)$ as the stationary distribution of the information state, because P(Z) remains unchanged in the time evolution, i.e., $P\left(Z^{\prime }\right)=\sum_{Z}P\left(Z^{\prime }|Z\right)P\left(Z\right)$.

It is noteworthy that, due to the memoryless nature of the channel, the information dynamics can be governed in terms of how the input and output letters, $x_{t}$ and $y_{t}$, evolve in time. Both $x_{t}$ and $y_{t}$ can be regarded as the block codes of length N=1. The transition probability in Equation (8) for the block can be reduced into the transition probability for the letter. By introducing the information state for the letter $z_{t}=\left(x_{t},y_{t}\right)$, the transition probability for the letter $z_{t}$ is given by

(10) $$\begin{array}{c} P\left(z_{t}|z_{t-1}\right)=Q\left(x_{t}\right)q\left(y_{t}|x_{t-1}\right). \end{array}$$

Consequentially, the Markovian information dynamics for the letter can be derived from the information dynamics for the block in Equation (9):

(11) $$\begin{array}{c} P\left(z_{t}\right)=\sum_{z_{t-1}}P\left(z_{t}|z_{t-1}\right)P\left(z_{t-1}\right). \end{array}$$

Then, $P\left(z\right)=P\left(z_{t\to \infty }\right)=Q\left(x\right)P\left(y\right)$ with $P\left(y\right)=\sum_{x}Q\left(x\right)q\left(y|x\right)$ is the stationary distribution of the information state *z*.

The transition probability $P(Z_{t}|Z_{t-1})$ in Equation (8) is recognized as the information driving force [24,25,26] behind the information dynamics for the block because $P(Z_{t}|Z_{t-1})$ transforms an information state $Z_{t-1}$ to another state $Z_{t}$ in time and determines the stationary distribution P(Z). For the same reason, the transition probability $P(z_{t}|z_{t-1})$ in Equation (11) can be treated as the information driving force for the letter or block length N=1.

### 3.2. Characterization of Nonequilibrium

Due to the exchange of energy and information between a system and its environment, the classical information channels should be considered as a nonequilibrium system. In particular, in ref. [42], a classical measurement model was developed to explore nonequilibrium phenomena of the information dynamics. It is shown that the Markov dynamics governing sequential measurements is governed by an information driving force that can be decomposed into two components: an equilibrium component that maintains time reversibility and a nonequilibrium component that violates time reversibility. In this work, we will examine the information dynamics of noise channels using this nonequilibrium framework.

The nonequilibrium nature is reflected in the time-irreversible behavior of the information dynamics. The time irreversibility means that the probability of a time sequence of the information state $\Omega =\left\{Z_{1},Z_{2},\cdots ,Z_{t}\right\}=\left\{\left(X_{1},Y_{1}\right),\left(X_{2},Y_{2}\right),\cdots ,\left(X_{t},Y_{t}\right)\right\}$ is different from the probability of the corresponding time-reversal sequence $\tilde{\Omega }=\left\{Z_{t},Z_{t-1},\cdots ,Z_{1}\right\}=\left\{\left(X_{t},Y_{t}\right),\left(X_{t-1},Y_{t-1}\right),\cdots ,\left(X_{1},Y_{1}\right)\right\}$. In the Markovian information dynamics given by Equation (9), the output $Y_{t}$ only depends on the previous input $X_{t-1}$ in the forward in time transition. On the other hand $Y_{t}$ is only determined by the “future” input $X_{t+1}$ in the backward in time transition. Furthermore, $X_{t}$ together with $Y_{t-1}$ and $Y_{t+1}$ has no influence on $Y_{t}$ in both time directions, and can be neglected in both transitions. Therefore, the time-irreversibility can be characterized by the time-irreversible information flux, which is defined as the difference between the probability of the transition from $X_{t-1}$ to $Y_{t}$ forward in time and the probability from $X_{t+1}$ to $Y_{t}$ backward in time.

By using Equation (9), we can define the information flux as

(12) $$\begin{array}{ccc} J_{Y_{t}}\left(X_{t-1},X_{t+1}\right) & = & \sum_{X_{t},Y_{t-1},Y_{t+1}}\left[P\left(Z_{t-1}\right)Q\left(Z_{t}|Z_{t-1}\right)-P\left(Z_{t+1}\right)Q\left(Z_{t}|Z_{t+1}\right)\right]\\ & = & 2Q_{N}\left(X_{t-1}\right)Q_{N}\left(X_{t+1}\right)d_{Y_{t}}\left(X_{t-1},X_{t+1}\right), \end{array}$$

with

(13) $$\begin{array}{c} d_{Y_{t}}\left(X_{t-1},X_{t+1}\right)=\frac{1}{2}\left[q_{N}\left(Y_{t}|X_{t-1}\right)-q_{N}\left(Y_{t}|X_{t+1}\right)\right]. \end{array}$$

Here, the blocks $X_{t}$, $Y_{t-1}$, and $Y_{t+1}$ are summed away from the probabilities because they are not important to the quantification of the time-irreversibility. We see that when the input distribution $Q_{N}\left(X\right)$ or Q(x) is given, the information flux *J* in Equation (12) merely depends on the difference between the transmission probabilities of the channel which is quantified by *d* in Equation (13). Then, *d* describes the strength of the information flux in this situation.

In particular, for the letter case where block length N=1, the information flux with the strength *d* can be given by the transmission probability q(y|x) according to Equations (12) and (13):

(14) $$\begin{array}{ccc} J_{y}\left(x_{t-1},x_{t+1}\right) & = & 2Q\left(x_{t-1}\right)Q\left(x_{t+1}\right)d_{y_{t}}\left(x_{t-1},x_{t+1}\right),\\ d_{y_{t}}\left(x_{t-1},x_{t+1}\right) & = & \frac{1}{2}\left[q\left(y_{t}|x_{t-1}\right)-q\left(y_{t}|x_{t+1}\right)\right]. \end{array}$$

### 3.3. Nonequilibrium Decomposition for Transmission Probability

The information flux *J* can be used to characterize the time-irreversibility because it is originated from the nonequilibrium of the transition probability or information driving force $P(Z_{t}|Z_{t-1})$ given by Equation (8). To show this point more explicitly, we firstly decompose $P(Z_{t}|Z_{t-1})$ into two parts [23,28],

(15) $$\begin{array}{c} P\left(Z_{t}|Z_{t-1}\right)=P_{m}\left(Z_{t-1},Z_{t},Z_{t+1}\right)+P_{d}\left(Z_{t-1},Z_{t},Z_{t+1}\right), \end{array}$$

with

(16) $$\begin{array}{c} \left\{\begin{array}{c} P_{m}\left(Z_{t-1},Z_{t},Z_{t+1}\right)=\frac{1}{2}\left[P\left(Z_{t}|Z_{t-1}\right)+P\left(Z_{t}|Z_{t+1}\right)\right]=Q_{N}\left(X_{t}\right)m_{Y_{t}}\left(X_{t-1},X_{t+1}\right),\\ P_{d}\left(Z_{t-1},Z_{t},Z_{t+1}\right)=\frac{1}{2}\left[P\left(Z_{t}|Z_{t-1}\right)-P\left(Z_{t}|Z_{t+1}\right)\right]=Q_{N}\left(X_{t}\right)d_{Y_{t}}\left(X_{t-1},X_{t+1}\right),\\ m_{Y_{t}}\left(X_{t-1},X_{t+1}\right)=\frac{1}{2}\left[q_{N}\left(Y_{t}|X_{t-1}\right)+q_{N}\left(Y_{t}|X_{t+1}\right)\right]. \end{array}\right. \end{array}$$

where *d* is given by Equation (13). Then, from the Markovian nature of the information dynamics, the probability of a time sequence $\Omega =\{Z_{1},Z_{2},\cdots ,Z_{t}\}$ and that of the corresponding time-reversal sequence $\tilde{\Omega }=\left\{Z_{t},Z_{t-1},\cdots ,Z_{1}\right\}$ can be given by Equation (9) as follows,

(17) $$\begin{array}{c} \left\{\begin{array}{cc} P(\Omega ) & =P\left(Z_{1}\right)\prod_{i=2}^{t}P\left(Z_{i}|Z_{i-1}\right)\\ & =P\left(Z_{1}\right)Q_{N}\left(X_{t}\right)P\left(Z_{t}|Z_{t-1}\right)\\ & \times \prod_{i=2}^{t-1}Q_{N}\left(X_{i}\right)\left[P_{m}\left(Z_{i-1},Z_{i},Z_{i+1}\right)+P_{d}\left(Z_{i-1},Z_{i},Z_{i+1}\right)\right],\\ P(\tilde{\Omega }) & =P\left(Z_{t}\right)\prod_{i=2}^{t}P\left(Z_{t-i+1}|Z_{t-i+2}\right)\\ & =P\left(Z_{t}\right)Q_{N}\left(X_{1}\right)P\left(Z_{1}|Z_{2}\right)\\ & \times \prod_{i=2}^{t-1}Q_{N}\left(X_{i}\right)\left[P_{m}\left(Z_{t-i+2},Z_{t-i+1},Z_{t-i}\right)+P_{d}\left(Z_{t-i+2},Z_{t-i+1},Z_{t-i}\right)\right)]. \end{array}\right. \end{array}$$

Now, we can discuss the relation between the time reversibility and the driving force of the information dynamics. When P(Ω) is equal to $P(\tilde{\Omega })$ for arbitrary possible time sequence *Z*, the information dynamics is under equilibrium state so that the time-reversal symmetry of each time sequence is preserved and the dynamics is time-reversible. From Equations (12) and (13), we can observe that $P\left(\Omega \right)=P\left(\tilde{\Omega }\right)$ for arbitrary possible time sequence *Z* is satisfied if and only if all the possible $P_{d}\left(Z_{i-1},Z_{i},Z_{i+1}\right)=0$ or $q_{N}\left(Y_{t}|X_{t-1}\right)=q_{N}\left(Y_{t}|X_{t+1}\right)$ holds for arbitrary $Y_{t}$, $X_{t-1}$ and $X_{t+1}$. In this situation, $P_{m}\left(Z_{i-1},Z_{i},Z_{i+1}\right)$ does not depend on any input block *X*. One can denote $P_{m}\left(Z_{i-1},Z_{i},Z_{i+1}\right)=P_{N}\left(Y_{i}\right)$ and $P\left(\Omega \right)=P\left(\tilde{\Omega }\right)=\prod_{i=1}^{t}Q_{N}\left(X_{i}\right)P_{N}\left(Y_{i}\right)$. Therefore, $P_{m}$ preserves the time-reversal symmetry and the “equilibrium” of the information dynamics. Then, $P_{m}$ is recognized as the equilibrium driving force in the information dynamics.

If $P_{d}\left(Z_{i-1},Z_{i},Z_{i+1}\right)\neq 0$, the information dynamics is time-irreversible and the channel is under nonequilibrium state. This is to say that the driving force $P_{d}$ breaks the time-reversal symmetry of the dynamics, and drives the dynamics away from the equilibrium state. Then, $P_{d}$ is recognized as the nonequilibrium driving force of the information dynamics.

Combining Equation (12) with Equation (16), the information flux can be rewritten as

$$\begin{array}{c} J_{Y}\left(X_{t-1},X_{t+1}\right)=\frac{2Q\left(X_{t-1}\right)Q\left(X_{t+1}\right)}{Q_{N}\left(X_{t}\right)}P_{d}\left(Z_{t-1},Z_{t},Z_{t+1}\right). \end{array}$$

This equation shows the relation between the information flux *J* and the nonequilibrium driving force $P_{d}$. Therefore, the information flux can also be used to characterize the time-irreversibility. On the other hand, the information flux *J* can be treated as the nonequilibrium driving force since it shares the same factor *d* with the nonequilibrium driving force $P_{d}$ as shown in Equation (12). For this reason, the strength of the information flux *J* also works as the strength of the nonequilibrium driving force (or simply as the nonequilibrium strength).

According to the discussion in the above, it can be seen that the information dynamics in Equation (9) is under equilibrium state if and only if d=0 for arbitrary input and output blocks *X* and *Y*. This leads to the detailed balance or equilibrium condition of the information dynamics,

(18) $$\begin{array}{c} d_{Y_{t}}\left(X_{t-1},X_{t+1}\right)=0,\;\mathrm{or}\;q_{N}\left(Y_{t}|X_{t-1}\right)=q_{N}\left(Y_{t}|X_{t+1}\right)\;\mathrm{for}\;\mathrm{all}\;X\;\mathrm{and}\;Y. \end{array}$$

If the detailed balance condition is violated, the dynamics is under nonequilibrium state. Then, the absolute value |d| characterizes the degree of the detailed balance breaking and the nonequilibrium of the dynamics.

On the other hand, the entity $m_{Y_{t}}\left(X_{t-1},X_{t+1}\right)$ given by Equation (16) works as the strength of the equilibrium driving force $P_{m}$ (or simply as the equilibrium strength). Due to the time-reversal symmetry of *m*, it preserves the detailed balance condition in Equation (18). Furthermore, both *m* and *d* merely depend on the transmission probability of the channel. Thus whether the information dynamics in equilibrium or not is totally determined by the channel itself.

There is a deep connection between the nonequilibrium in information transmission and the performance of the error correction. As a glimpse of this connection, we can see that the error correction given achieves the worst performance, which means that the error occurs with probability 1, if and only if the information dynamics achieves the equilibrium condition in Equation (18). This is because the input *X* and output *Y* are totally independent of each other ($q_{N}\left(Y|X\right)=q_{N}\left(Y|X^{\prime }\right)$), and there is no useful information transferred during the information dynamics. Then, arbitrary two transmitted symbols $s^{\prime }$ and *s* cannot be distinguished by the decoder anymore. Otherwise, the nonequilibrium characterized by the non-vanishing nonequilibrium strength *d* can improve the performance of the error correction, which will be discussed in detail in the following.

### 3.4. Nonequilibrium Decomposition for Transmission Probability

There is a relation between the information driving force $P(Z_{t}|Z_{t-1})$ and the transmission probability $q_{N}\left(Y|X\right)$, which is given by Equation (8). The decomposition of information driving force $P(Z_{t}|Z_{t-1})$ in Equations (15) and (16) suggests a decomposition of transmission probability $q_{N}\left(Y|X\right)$ in the form of

(19) $$\begin{array}{c} q_{N}\left(Y|X\right)=m_{Y}\left(X,X^{\prime }\right)+d_{Y}\left(X,X^{\prime }\right),\;\mathrm{for}\;X^{\prime }\neq X \end{array}$$

with

(20) $$\begin{array}{c} \left\{\begin{array}{c} d_{Y}\left(X,X^{\prime }\right)=\frac{1}{2}\left[q_{N}\left(Y|X\right)-q_{N}\left(Y|X^{\prime }\right)\right]\\ m_{Y}\left(X,X^{\prime }\right)=\frac{1}{2}\left[q_{N}\left(Y|X\right)+q_{N}\left(Y|X^{\prime }\right)\right]. \end{array}\right. \end{array}$$

Here, the two parts *m* and *d* given in Equation (20) correspond to the equilibrium and nonequilibrium strengths in the nonequilibrium information dynamics, respectively.

In particular, the nonequilibrium decomposition for $q_{N}\left(Y|X\right)$ can be also applied to the transmission probability q(y|x) for the letters. The explicit form is then given by

(21) $$\begin{array}{c} q\left(y|x\right)=m_{y}\left(x,x^{\prime }\right)+d_{y}\left(x,x^{\prime }\right),\;\mathrm{for}\;x^{\prime }\neq x \end{array}$$

with

(22) $$\begin{array}{c} \left\{\begin{array}{c} d_{y}\left(x,x^{\prime }\right)=\frac{1}{2}\left[q\left(y|x\right)-q\left(y|x^{\prime }\right)\right]\\ m_{y}\left(x,x^{\prime }\right)=\frac{1}{2}\left[q\left(y|x\right)+q\left(y|x^{\prime }\right)\right]. \end{array}\right. \end{array}$$

Due to the decomposition in Equations (19) and (20), both *m* and *d* can change independently within the properly given ranges of the constraints. Consequentially, $q_{N}\left(Y|X\right)$ can be changed by altering *m* and *d*. This can improve the performance of the error correction as discussed in the above. Due to the nonnegativity and normalization of the transmission probabilities q(x|s), the constraints on *m* and *d* can be given as follows,

(23) $$\begin{array}{c} \left\{\begin{array}{c} 0\leq q_{N}\left(Y|X\right)=m_{Y}\left(X,X^{\prime }\right)+d_{Y}\left(X,X^{\prime }\right)\leq 1\\ 0\leq m_{Y}\left(X,X^{\prime }\right)\leq 1\\ \sum_{Y}m_{Y}\left(X,X^{\prime }\right)+d_{Y}\left(X,X^{\prime }\right)=\sum_{Y}q_{N}\left(Y|X\right)=1 \end{array}\right., \end{array}$$

The second constraint is originated from nonnegativity of the transmission probability $q_{N}\left(Y|X\right)$, because $0\leq m_{Y}\left(X,X^{\prime }\right)=\frac{1}{2}\left[q_{N}\left(Y|X\right)+q_{N}\left(Y|X^{\prime }\right)\right]\leq 1$.

It is important to observe that when the equilibrium strength *m* is fixed at each block code *X* and *Y*, the nonequilibrium strength *d* can form a convex set.

$$\begin{array}{c} D_{N}=\left\{d:d_{Y}\left(X,X^{\prime }\right)\;\mathrm{satisfies}\;\mathrm{the}\;\mathrm{constraints}\;\mathrm{in}\;\mathrm{Equation}\;\left(23\right)\right\}\;. \end{array}$$

The convexity of this set can be easily observed as follows. As shown in the proof of the concavity of the upper bound in Equation (7), the transmission probabilities $q_{N}\left(Y|X\right)=m_{Y}\left(X,X^{\prime }\right)+d_{Y}\left(X,X^{\prime }\right)$ form a convex set $Q_{N}$. If *m* is fixed in $q_{N}\left(Y|X\right)$, then *d* becomes the affine transform of $q_{N}\left(Y|X\right)$ in the form of $d_{Y}\left(X,X^{\prime }\right)=q_{N}\left(Y|X\right)-m_{Y}\left(X,X^{\prime }\right)$ (here $m_{Y}\left(X,X^{\prime }\right)$ becomes a constant), which transforms the elements in $Q_{N}$ into $D_{N}$. Since $Q_{N}$ is a convex set and the affine transform always preserves the convexity [41], then $D_{N}$ is also a convex set.

Although there exist large numbers of the decompositions of the transmission probabilities $q_{N}\left(Y|X\right)$ in the form of Equation (19) corresponding to different block lengths *N*, the decomposition in Equation (21), which corresponds to the letters or the block length of N=1, gives a fundamental description of this nonequilibrium information dynamics. This is because not only every input block code is composed of the letters *x* which are distorted independently into the output letters *y*, but also the detailed balance condition (Equation (18)) for the information dynamics at block lengths of *N* (Equation (9)) is determined by the detailed balance condition for the information dynamics at block lengths of N=1 (Equation (11)). This can be seen from Equation (1) that $q_{N}\left(Y|X\right)=q_{N}\left(Y|X^{\prime }\right)$ for arbitrary input block $X^{\prime }\neq X$ if and only if every $q\left(y|x\right)=q\left(y|x^{\prime }\right)$ or $d_{y}\left(x,x^{\prime }\right)=0$ for all $x^{\prime }\neq x$, which is exactly the detailed balance condition for the block length of N=1. In other words, if the information dynamics for letters in Equation (11) achieves the equilibrium, then every information dynamics for the block codes achieves the equilibrium. Furthermore, the absolute value of the nonequilibrium strength |d| for the letters given in Equation (22) characterizes the degree of the nonequilibrium of the memoryless channel model.

## 4. Nonequilibrium Information Thermodynamics and Performance of Error Correction

### Entropy Production Rate Guarantees Reliability of Information Transmission

It is well known that the nonequilibrium in a system can give rise to thermodynamic dissipation cost from the net input energy, matter, or information. The dissipation cost can be properly quantified by the entropy production rate (EPR). It is defined as the log ratio between the probability of the time sequence $\Omega =\{Z_{1},Z_{2},\cdots ,Z_{t}\}$ and that of its time-reversal sequence $\tilde{\Omega }=\left\{Z_{t},Z_{t-1},\cdots ,Z_{1}\right\}$, by taking the average over all the possible sequences and over time [28],

(24) $$\begin{array}{c} \mathrm{EPR}=\frac{1}{t}\sum_{\Omega }P\left(\Omega \right)\log\frac{P(\Omega )}{P(\tilde{\Omega })}\geq 0,\;\mathrm{for}\;\mathrm{large}\;t \end{array}$$

Here, the process is supposed to be stationary and ergodic.

Mathematically, the EPR is in the form of the relative entropy $D_{KL}(P\left(\Omega \right)||P\left(\tilde{\Omega }\right))$ between the distributions P(Ω) and $P(\tilde{\Omega })$. Thus, EPR is always nonnegative due to the nonnegativity of the relative entropy. In particular, EPR =0 if and only if the equality $P\left(\Omega \right)=P\left(\tilde{\Omega }\right)$ holds for all the possible Ω and $\tilde{\Omega }$. This indicates that the system dynamics is time-reversible. Otherwise, the system dynamics is time-irreversible for EPR >0 [27,36].

Physically, the term $r=\frac{1}{t}\log\frac{P(\Omega )}{P(\tilde{\Omega })}$ quantifies the rate of the stochastic entropy change in both the system and the environments [28,37]. The stochastic entropy change *r* fluctuates along with the time sequences and can be either positive or negative. However, the average of *r* over the ensemble of the system, which is the EPR exactly, follows the fluctuation theorem and becomes nonnegative. This can give rise to the second thermodynamic law that the (averaged) entropy change in both the system and the environments can never decrease. The vanishing EPR indicates that the system is at the equilibrium state and there is no net exchange between the system and the environments on average. Otherwise, the system is at nonequilibrium state with an inevitable thermodynamic dissipation from the system to the environments quantified by nonzero EPR.

We are interested in the EPR because it is not only the quantification of the thermodynamic dissipation cost but is also a proper description of the reliability of the information transmission. To see this, we evaluate the EPR of the information dynamics in Equation (9) by substituting the probabilities of the time sequence Ω and its time-reversal sequence $\tilde{\Omega }$ in Equation (17) into the expression of the EPR in Equation (24). This yields [25,26],

(25) $$\begin{array}{ccc} \mathrm{EPR} & = & \frac{1}{2}\sum_{X_{i-1},X_{i+1},Y_{i}}J_{Y_{i}}\left(X_{i-1},X_{i+1}\right)\log\frac{q_{N}\left(Y_{i}|X_{i-1}\right)}{q_{N}\left(Y_{i}|X_{i+1}\right)}\\ & = & \sum_{X,X^{\prime },Y}Q_{N}\left(X\right)Q_{N}\left(X^{\prime }\right)d_{Y}\left(X,X^{\prime }\right)\log\frac{q_{N}\left(Y|X\right)}{q_{N}\left(Y|X^{\prime }\right)}\\ & = & N\sum_{x,x^{\prime },y}Q\left(x\right)Q\left(x^{\prime }\right)d_{y}\left(x,x^{\prime }\right)\log\frac{m_{y}\left(x,x^{\prime }\right)+d_{y}\left(x,x^{\prime }\right)}{m_{y}\left(x,x^{\prime }\right)-d_{y}\left(x,x^{\prime }\right)}\geq 0 \end{array}$$

The first equality in Equation (25) expresses the EPR by using the micro states with the perspective of the nonequilibrium information dynamics. Note that the information flux *J* is provided by Equation (12). One can define *l* that quantifies the detailed information loss during the transition in the channel as follows

$$\begin{array}{c} l=\log\frac{q_{N}\left(Y_{i}|X_{i-1}\right)}{q_{N}\left(Y_{i}|X_{i+1}\right)}\;. \end{array}$$

This term *l* is in the first equality in Equation (25). It can also be given by the information difference as $l=I\left(X,Y\right)-I\left(X^{\prime },Y\right)$. Here, the information *I* refers to the reduction in the uncertainty of the output block *Y* caused by the input block *X*, quantified by the difference between the prior uncertainty $-\log P_{N}\left(Y\right)$ and the posterior uncertainty $-\log q_{N}\left(Y|X\right)$ as

$$\begin{array}{c} I\left(X,Y\right)=\log\frac{q_{N}\left(Y|X\right)}{P_{N}\left(Y\right)}\;. \end{array}$$

Intuitively, if the input *X* reduces more uncertainty in the output *Y* compared to another input $X^{\prime }$, then *Y* carries more information of *X* than $X^{\prime }$, and consequentially the transmission probability $q_{N}\left(Y|X\right)>q_{N}\left(Y|X^{\prime }\right)$.

For no loss of the generality, let us consider the following case. It is possible that the input blocks at time i−1 and at time i+1 can be *X* and $X^{\prime }\neq X$, respectively, but these two inputs can appear at $i^{\prime }-1$ and at $i^{\prime }+1$ in a time-reversed order. Thus $\left\{X_{i^{\prime }-1}=X^{\prime },X_{i^{\prime }+1}=X\right\}$ can be regarded as the time-reversal of the sequence $\left\{X_{i-1}=X,X_{i+1}=X^{\prime }\right\}$. However, due to the randomness of the channel, the same output *Y* can be originated both from *X* and $X^{\prime }$, and can be received at time *i* and $i^{\prime }$, respectively. Assume that *Y* carries more information of *X* than $X^{\prime }$. When *Y* appears to be from *X* but is actually from $X^{\prime }$, then the information of *X* is lost with the quantification $l=I\left(X,Y\right)-I\left(X^{\prime },Y\right)>0$. Here, the nonnegativity of information loss can be guaranteed by the symmetry in the EPR, because l>0 while the information flux J>0; otherwise, l<0 at J<0. Thus, either l<0 or l>0 can be regarded as the positive information loss.

Driven by the non-vanishing information flux J≠0 under nonequilibrium, the information loss or dissipation can be positive. Otherwise, the vanishing information flux *J* leads to zero information dissipation under equilibrium. Since the term *l* is the difference in logarithm of the probability, one can regard the information loss as the analog of voltage (potential related to the population) difference while the information flux as the current in electronic circuit. The entropy production rate is then the analog of the power generated or cost in the electric circuit. This gives the physical interpretation of the first equality in Equation (25).

The second equality in Equation (25) expresses the EPR as a quantification of the reliability of the information transmission, with the nonequilibrium strength of the information flux and the information driving force *d* provided in Equations (19) and (20). Note that the time in the subscript of every block in the first equality in Equation (25) is no longer important in the second equality any more.

To see the EPR as the quantification of reliability, an easy-to-understand explanation is that if the peaks of two transmission probability distributions $q_{N}$, conditioning on the inputs *X* and $X^{\prime }$, respectively, do not coincide with the same outputs. Then, the corresponding symbols are transmitted with less random interference in the channel, and the decoder can more easily distinguish between symbols. Conversely, if the peak positions of two transmission probability distributions overlap, it is difficult to decode the corresponding symbols correctly. The distance between two transmission probability distributions can be used to measure the difference between their peak positions, and the EPR is an appropriate tool for this measurement.

The third equality in Equation (25) expresses the EPR or the reliability of the information transmission in terms of the nonequilibrium decomposition of the transmission probability q(y|x) for the letters given by Equations (21) and (22). This expression arises from the memoryless nature of the channel, and then the nonequilibrium of the transmission probability q(y|x) becomes significantly important to the information dynamics. Consequentially, the nonequilibrium part $d_{y}\left(x,x^{\prime }\right)$ in q(y|x) determines the nonequilibrium of the dynamics. As a function of the nonequilibrium strength *d*, the EPR is convex on the convex set D of *d*, with the equilibrium strength *m* and the input distribution Q(x) being fixed. The convexity of D has been proved in Section 3.3 (D = $D_{N}$ for N=1). Now, let us prove the convexity of the EPR in the following way.

Choose arbitrary two $d^{\left(1\right)},d^{\left(2\right)}\in D$. The corresponding convex combination of the EPRs with respect to $d^{\left(1\right)}$ and $d^{\left(2\right)}$ can be given by

(26) $$\begin{array}{c} \;\;\;\;\;\;\;\;\lambda \mathrm{EPR}\left(d^{\left(1\right)}\right)+\left(1-\lambda \right)\mathrm{EPR}\left(d^{\left(2\right)}\right)\\ =\;\;\lambda N\sum_{x,x^{\prime },y}Q\left(x\right)Q\left(x^{\prime }\right)d_{y}^{\left(1\right)}\left(x,x^{\prime }\right)\log\frac{m_{y}\left(x,x^{\prime }\right)+d_{y}^{\left(1\right)}\left(x,x^{\prime }\right)}{m_{y}\left(x,x^{\prime }\right)-d_{y}^{\left(1\right)}\left(x,x^{\prime }\right)}\\ +\;\;\left(1-\lambda \right)N\sum_{x,x^{\prime },y}Q\left(x\right)Q\left(x^{\prime }\right)d_{y}^{\left(2\right)}\left(x,x^{\prime }\right)\log\frac{m_{y}\left(x,x^{\prime }\right)+d_{y}^{\left(1\right)}\left(x,x^{\prime }\right)}{m_{y}\left(x,x^{\prime }\right)-d_{y}^{\left(2\right)}\left(x,x^{\prime }\right)}\\ =\;\;\mathrm{EPR}(\lambda d^{\left(1\right)}+\left(1-\lambda \right)d^{\left(2\right)}). \end{array}$$

Here, the log-sum inequality $\sum_{i}a_{i}\ln\frac{a_{i}}{b_{i}}\geq \left(\sum_{i}a_{i}\right)\ln\frac{\sum_{i}a_{i}}{\sum_{i}b_{i}}$ in [23] is applied in Equation (26) for λ∈[0,1]. Due to the convexity of D, the convex combination $\lambda d^{\left(1\right)}+\left(1-\lambda \right)d^{\left(2\right)}$ in Equation (26) is still in D. Thus, EPR(d) is convex on D according to the definition of the convex function. This completes the proof.

The convexity shown in Equation (6) indicates that larger EPR values correspond to larger nonequilibrium strengths or distances between transmission probability distributions. Thus, improving the condition of transmission can be achieved by increasing the energy cost (EPR) of information transmission, or by enlarging the nonequilibrium strength of the channel. It is noteworthy that, when considering a fixed equilibrium strength *m* and input distribution Q(x), the EPR achieves the unique minimum 0 if and only if the information dynamics in Equation (10) reaches equilibrium, i.e., d=0. This corresponds to the worst condition of transmission that all the symbols cannot be distinguishable at the decoder.

## 5. Nonequilibrium Dissipation Cost Improves Performance of Error Correction

The nonequilibrium energy cost quantified by EPR (Equation (25)) can improve information transmission by reducing the interference between symbols, and then can improve the error correction performance.

To see this, we note that the upper bound of the decoding error probability in Equation (5) quantifies the worst performance of the error correction. Since the nonequilibrium of the transmission probability q(y|x) is fundamental to the information dynamics, we use the nonequilibrium decomposition for q(y|x) Equations (21) and (22) and the upper bound for the memoryless channel in the second equality of Equation (6) to figure out the influence of the nonequilibrium in the information transmission.

One can observe that the function $u=\sum_{y}{\left[\sum_{x}Q\left(x\right){\left[q\left(y|x\right)\right]}^{\frac{1}{\rho }}\right]}^{\rho }$ in the upper bound of the error probability can be rewritten as the function of the nonequilibrium strength *d*. It is given by

(27) $$\begin{array}{c} u\left(d\right)=\sum_{y}{\left[\sum_{x}Q\left(x\right){\left[m_{y}\left(x,x^{\prime }\right)+d_{y}\left(x,x^{\prime }\right)\right]}^{\frac{1}{\rho }}\right]}^{\rho }. \end{array}$$

We have proven in Equation (7) that *u* is a concave function of q(y|x) with fixed equilibrium strength *m*, input distribution Q(x), and parameter ρ. Since the correspondence between q(y|x) and $d_{y}\left(x,x^{\prime }\right)$ is an affine transformation given by Equation (21), then the concavity of *u* is preserved by this affine transformation, i.e., *u* is a concave function of *d* with the unique maximum 1 at the equilibrium point of the information dynamics in Equation (10) where d=0. Otherwise, the function *u* decreases as each absolute value $|d_{y}\left(x,x^{\prime }\right)|$ increases.

Consequentially, the upper bound of the error probability, given as ${\left(M-1\right)}^{\rho }{\left[u\left(d\right)\right]}^{N}$, also achieves the unique maximum ${\left(M-1\right)}^{\rho }$ where d=0, and decreases monotonically as each absolute value $|d_{y}\left(x,x^{\prime }\right)|$ or the nonequilibrium in the information transmission increases. Thus, higher nonequilibrium decreases the error probability and thus improves the performance. In particular, when d=0, the equilibrium is reached, then the performance of the error correction is the worst. This also indicates that one needs to go to the nonequilibrium regime to improve the error corrections.

On the other hand, the EPR in Equation (25) can describe the reliability of the information transmission in the perspective of the nonequilibrium information dynamics. In addition, the EPR is directly related to the measure of the nonequilibrium characterized by *d*. Then, the performance of the error correction can also be a function of the EPR. We find that the performance of the error corrections becomes better with the decrease in the upper bound of the error correction when the EPR increases. Then, we can see that the dissipative cost can enhance the performance of the error corrections. This shows how nonequlibrium in transmission can help to improve the error correction in decoding from the thermodynamic perspective.

## 6. An Illustrative Case of Binary Memoryless Channel

In this section, we illustrate the above idea through analysing how the performance of error correction is influenced by the nonequilibrium in the binary memoryless channel model. In this example, all the entities that we have studied in the previous sections can be given in explicit forms and be interpreted clearly.

The transmitted symbols are simply assumed to be s=a,b. The encoder assigns binary block codes to the symbols such as a=000, b=111. For the simplicity of the encoder, let us use the random encoding method, with the input distribution for the encoding letters x=0,1 given by {Q(x=0)=p,Q(x=1)=1−p}.

The input block codes are interfered by the two random and independent noise sources which form the channel. The settings of this case are as follows. If the encoder generates the letters *x* independently and identically distributed with the input distribution Q(x) at time *t*, then the receiver receives the output symbols y=0,1 at t+1, which satisfy the following equation

(28) $$\begin{array}{c} y_{t+1}=\eta_{t}x_{t}+\xi_{t}\left(1-x_{t}\right), \end{array}$$

where η and ξ are the noises generated in the two noise sources, respectively, at that moment. The probabilities of the noises are given by

$$\begin{array}{c} P\left(\eta =0\right)=e_{1},P\left(\eta =1\right)=1-e_{1}\;,\\ P\left(\xi =0\right)=e_{2},P\left(\xi =1\right)=1-e_{2}\;. \end{array}$$

By using Equation (28), we obtain the correspondence between $x_{t}$ and $y_{t+1}$ under the noises (see Table 1). The noise *m* influences the input letter x=0, since $y_{t+1}=\xi_{t}$ when $x_{t}=0$. Meanwhile, the noise *n* distorts the letter x=1, since $y_{t+1}=\eta_{t}$ when $x_{t}=1$.

According to the description in the above, the transmission probabilities q(y|x) can be given by the probabilities of the noises as follows

(29) $$\begin{array}{c} q\left(y|x\right)=\left\{\begin{array}{cc} e_{1}, & y=0,x=0\\ e_{2}, & y=0,x=1\\ 1-e_{1}, & y=1,x=0\\ 1-e_{2}, & y=1,x=1 \end{array}\right. \end{array}$$

The difference between the transmission probabilities is recognized as the strength of the nonequilibrium part of the driving force or the time-irreversible information flux according to Equations (14) and (15), which is given by

(30) $$\begin{array}{c} d=\frac{1}{2}\left(e_{1}-e_{2}\right), \end{array}$$

and the strength of the equilibrium part of the driving force can be given by

(31) $$\begin{array}{c} m=\frac{1}{2}\left(e_{1}+e_{2}\right). \end{array}$$

The decomposition of the transmission probabilities in this case can be given by Equation (21) as

(32) $$\begin{array}{c} e_{1}=m+d,\;\mathrm{and}\;e_{2}=m-d. \end{array}$$

The constraints on *m* and *d* can be given by Equation (23) as

(33) $$\begin{array}{c} \left\{\begin{array}{c} 0\leq m+d\leq 1\\ 0\leq m-d\leq 1\\ 0\leq m\leq 1 \end{array}\right., \end{array}$$

Then, *d* can be chosen from the convex set D=[a,b] with fixed *m*, where the lower bound is $l_{d}=\max\left(m-1,-m\right)<0$ and the upper bound is $u_{d}=\min\left(1-m,m\right)$. More explicitly, the convex set is D=[−m,m] for 0<m≤1/2, and becomes D=[m−1,1−m] for 1/2<m<1. Here, for numerical illustration, we select four sets of equilibrium strengths *m* and input probabilities Q(x=0)=p which are shown in Table 2.

We show that more nonequilibrium or information flux leads to more effective error correction at the decoder. As both the quantifications of the thermodynamic dissipation cost and the reliability of the error correction, the EPR can be rewritten as the function of *d*, as |d| being the strength of the nonequilibrium driving force or the information flux characterizing the degree of the nonequilibrium. From Equation (25), the EPR is given by

$$\begin{array}{c} \mathrm{EPR}=2Np\left(1-p\right)d\log\frac{\left(m+d)(1-m+d\right)}{\left(m-d)(1-m-d\right)}. \end{array}$$

where *N* is the block length.

**Figure 1:**
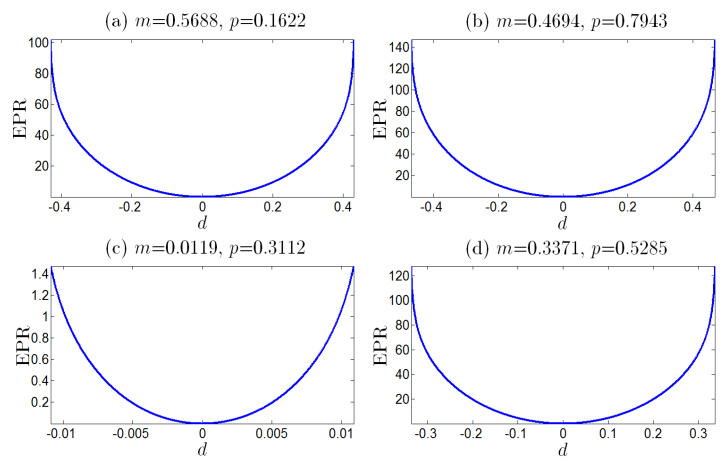
The EPR as the convex function of the nonequilibrium *d*, with fixed parameters N=100, *m* and *p* given in Table 2. The minima of all the EPRs are zero at the equilibrium point where d=0, and the EPRs increase monotonously as the absolute value |d| increases.

In Figure 1, the EPR as the function of *d* is potted for different *m* and *p*. It is shown that, for the fixed *m* and *p*, the EPR has a convex shape with a global minimum 0 at d=0. The minimum indicates that the reliability of the information transmission is zero at equilibrium. Otherwise, the EPR monotonically increases when the nonequilibrium or absolute value |d| increases (see Figure 1).

The performance of the error correction, which is described by the upper bound in Equation (5), has an intrinsic connection with the nonequilibrium in the information transmission characterized by *d* in Equation (21). By using Equation (27), we see that the upper bound of the error probability *G* is a simple function of *d*, which is explicitly given by

(34) $$\begin{array}{ccc} G & = & {\left(M-1\right)}^{\rho -1}U\left(Q,q_{N},\rho \right)\\ & = & {\left[p^{2}+{\left(1-p\right)}^{2}+2p\left(1-p\right)\left(\sqrt{m^{2}-d^{2}}+\sqrt{{\left(1-m\right)}^{2}-d^{2}}\right)\right]}^{N}. \end{array}$$

where the parameters are set as ρ=2 and M=2.

**Figure 2:**
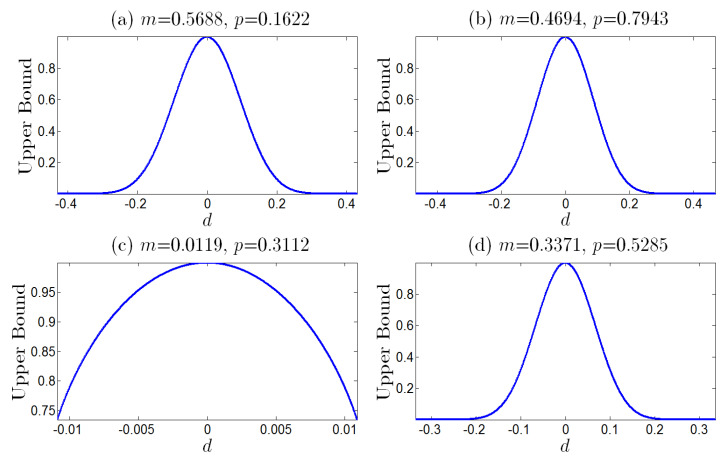
The upper bound of the error probability in the error correction as the function of the nonequilibrium *d*, with fixed parameters N=100, *m* and *p* given in Table 2. The maxima of all the upper bounds go to 1 at the equilibrium point where d=0. Correspondingly, the performance decreases monotonously as the absolute value |d| increases.

**Figure 3:**
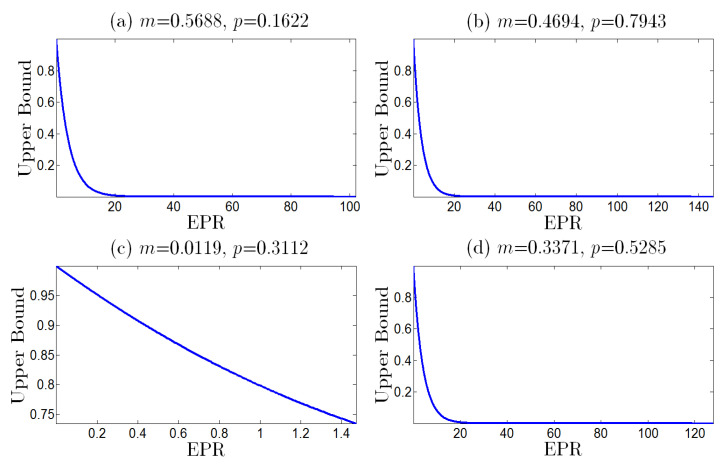
The upper bound of the error probability in the error correction as the function of the EPR, with fixed parameters N=100, *m* and *p* given in Table 2. The upper bound of the error probability decreases monotonously as the EPR increases.

In Figure 2, we have plotted the upper bound *G* as the function of *d* for different *m* and *p*. It can be easily verified that *G* monotonically decreases as the nonequilibrium |d| increases (see Figure 2). In addition, we also plot the upper bound *G* as the function of EPR in Figure 3, which shows that *G* monotonically decreases as the thermodynamic dissipation cost EPR increases. Therefore, we conclude that the larger absolute value of the asymmetry of the noise probability |d|, or the strength of nonequilibrium driving force (or the information flux), characterizing the larger nonequilibrium, results in the smaller error probability and the better performance of the error correction.

## 7. Conclusions

In this study, we aim to investigate the performance of error correction in information transmission. Our focus is on the memoryless channel model, and our findings suggest that nonequilibrium dynamics can enhance error correction performance. From a thermodynamic perspective, we demonstrate that increasing the nonequilibrium dissipation cost in the information transmission can lead to improved error correction performance of the decoder. These results may have broader implications beyond memoryless channels, and we plan to explore this in future studies.

The transfer of information is a critical process in biological systems, occurring in a variety of scenarios such as neural networks, DNA replication, and tRNA selection during translation. In molecular biology, the central dogma states that genetic information flows from DNA to RNA and from RNA to protein, with information referring to the precise determination of sequence for nucleic acid bases or amino acid residues in proteins [5]. Despite the stochastic nature of the underlying chemical processes, these information transfer processes require high accuracy. While kinetic proofreading is a well-known mechanism for error correction in biochemical reactions [6], the underlying mechanism for improving the performance of error corrections is still not fully understood, despite the existence of several classes of error-correction codes at the cellular level [7]. Our study reveals that the nonequilibrium effects resulting from dissipation cost to the environment are essential for improving the performance of error corrections, a finding with potential implications for investigating the universal mechanism by which information is transferred with high accuracy in biological systems. We plan to explore this further in future research.

## Figures and Tables

**Table 1:** Correspondence between the output $y_{t+1}$ and the input $x_{t}$ under noises.

$x_{t}$	0	0	0	0	1	1	1	1
$\eta_{t}$	0	0	1	1	0	0	1	1
$\xi_{t}$	0	1	0	1	0	1	0	1
$y_{t+1}$	0	1	0	1	0	0	1	1

**Table 2:** Sets of fixed equilibrium strength *m* and input probability Q(x=0)=p used for numerical illustrations.

	Set (a)	Set (b)	Set (c)	Set (d)
*m*	0.5688	0.4694	0.0119	0.3371
*p*	0.1622	0.7943	0.3112	0.5285

## Data Availability

Data sharing is not applicable to this article as no datasets were generated or analyzed during the current study.
